# Tailoring the Structural, Optical, and Electrical Performance of PVA Through Sn_0.8_Mn_0.2_S Nanostructures Incorporation for Optoelectronic Applications

**DOI:** 10.3390/polym18202577

**Published:** 2026-10-19

**Authors:** Mohammed G. Althobaiti

**Affiliations:** Department of Physics, College of Science, Taif University, P.O. Box 11099, Taif 21944, Saudi Arabia; m.althobaiti@tu.edu.sa

**Keywords:** PVA nanocomposite, Sn_0.8_Mn_0.2_S nanostructures, optical bandgap, refractive index, dielectric constant, electrical conductivity

## Abstract

This work aims to produce a new nanocomposite of Sn_0.8_Mn_0.2_S nanostructures incorporated into polyvinyl alcohol (PVA) for optoelectronic applications. Sn_0.8_Mn_0.2_S/PVA nanocomposite was prepared by combining a precipitation-based synthesis of Sn_0.8_Mn_0.2_S with the solution casting of the PVA matrix. FTIR spectroscopy was used to examine the chemical interactions and bonding between the Sn_0.8_Mn_0.2_S nanostructures and PVA chains. Morphological properties were investigated by the SEM technique. The X-ray diffraction technique was used to study the structural properties. UV–Visible spectroscopy was applied to explore the optical properties. Optical analysis shows that the direct and indirect optical bandgap decreased from 5.36 and 4.95 eV for the plain PVA to 1.94 and 1.32 eV at the highest loading (10 wt.%), accompanied by an increase in the refractive index from 1.69 to 2.33. Both the dielectric constants and optical conductivity of PVA likewise enhanced markedly with an increasing Sn_0.8_Mn_0.2_S amount. Four-probe DC measurements performed across 300 to 400 K demonstrated a reduction in PVA resistivity together with a drop in the activation energy, from 0.39 eV down to 0.18 eV, as the loading increased up to 1.0 wt.%, identifying this concentration as the most favorable for electrical performance. These findings recommend Sn_0.8_Mn_0.2_S nanostructures as an effective means of engineering the structural, optical, and electrical characteristics of PVA, with a clear relevance to optoelectronic device applications.

## 1. Introduction

Polymer nanocomposites (PNCs) have been attracting increasing interest in the field of materials research due to the wide range of physical, chemical, and biological properties [1,2,3,4,5]. These features make them an attractive fit for a lot of applications in industry, medicine, entertainments, and transportation [5,6,7,8]. Polymers can be processed to be either electrical insulators or conductors under specific conditions. Of these polymers, polyvinyl alcohol (PVA) stands out as non-toxic, water-soluble, biocompatible, and having semi-crystalline features [9,10,11]. The optical energy gap of PVA films is relatively large (~5.4 eV [5]), which results in a high transmittance in the visible and near-infrared regions, making the products suitable hosts for a variety of fillers. These physical characteristics qualify PVA nanocomposites for various optical and photonic applications [12,13]. Moreover, the hydroxyl (OH) groups along the backbone of PVA offer sites for successful reactions and forming stable PNCs. For example, Alharthi and Badawi produced ZrO_2_-SiC-doped PVA/rGO films for optical and X-ray shielding applications [14]. The Alziyadi group investigated the impact of PbI_2_ filling on PVA’s thermal and optical characteristics [15]. Ragab et al. [16] reinforced the electrical and optical characteristics of the PVA–PEO blend through graphene and MWCNT incorporation. The dielectric and radiation shielding of the PVP–PVA composite were significantly enhanced by the Mohamed group via SnS_2_ nanostructures [11]. The optoelectrical and antibacterial performance of the electrospum PVA–PVP–PAAm blend was enhanced by Rasheed et al. through TiO_2_ nanostructures for medical device applications [17]. Marashdeh et al. [18] showed that Bi_2_O_3_-nanostructure-doped PVA/PVC films are potential candidates in γ-ray shielding applications. Nd_2_O_3_ nanostructures were utilized by Soliman et al. [10] as an active filler in a PVA–MWCNT blend for optical applications. In our previous work [3], the radiation shielding of the PVA–rGO blend was effectively enriched through metal oxide filling. The silver-nanostructure-filled PVA composite was used by Iswarya et al. [19] as a solid polymer electrolyte. The graphene–PVA composite was applied for photovoltaic cell and diode applications by Alhuthali et al. [20]. Alsulami and Rajeh used a GO/Fe_3_O_4_ composite as an active filler in a PVA–CMC blend for energy storage applications [21]. Recent work on electrospun PVA–PVP nanofibers incorporated with TiO_2_ NPs shows that the optical bandgap of the prepared nanofibers decreased significantly, with a parallel enhancement in the electrical conductivity [22].

Compared to the other fillers, metal sulfides have many advantages such as their thermal and chemical stability, and high electrical and mechanical performance [23]. Tin sulfide (SnS) is a low-cost, environmentally friendly, and non-toxic semiconducting material. It possesses a narrow bandgap (E_g_~1.3 eV [24]) with a large optical absorption coefficient (~10^5^ cm^−1^). These characteristics make it a potential choice for plenty of applications [5,25,26]. Parallelly, manganese sulfide (MnS) has been found to be a wide-gap semiconductor (~3.7 eV [27]) with low toxicity and strong magnetic dilution, which has been applied to optical data storage, mass memory devices, and supercapacitor electrodes [28,29,30]. Mixing Sn and Mn sulfides can be a pathway to producing Sn_1−x_Mn_x_S nanostructures with an intermediate and composition-tunable bandgap, which are not easily achieved with either binary one. It places the resulting ternary bandgap between the two binary limits (SnS~1.3 eV and MnS~3.7 eV) while keeping Sn as the majority cation, which is expected to preserve the higher optical absorption coefficient and lower resistivity characteristic of SnS, whereas the benefiting from the low toxicity and magnetic dilution is offered by the MnS component. A moderate (20%) Mn substitution was selected to introduce these features without excessively widening the bandgap back toward the MnS limit, in line with the aim of achieving a strongly tunable, narrow-gap PVA nanocomposite for optoelectronic use. This, in principle, suggests Sn_0.8_Mn_0.2_S nanostructures (NSs) as a filler for PVA, because its physicochemical properties of the resulting nanocomposite can be simply adjusted via controlling the filler’s concentration.

The present work aims to produce Sn_0.8_Mn_0.2_S nanostructures incorporated PVA films for optoelectronic applications. Sn_0.8_Mn_0.2_S nanostructures were prepared by the precipitation method and incorporated in PVA with a variety of different wt.% (0.1, 0.5, 1.0, 5.0, and 10.0) by the solution casting method. FTIR and X-ray diffraction were used to elucidate the structural changes. SEM investigations were made to examine the morphological characteristics of the prepared samples. The UV–Visible spectroscopy technique was employed to characterize the optical properties of the films, and the optical bandgap was considered by both the Tauc and the absorption-coefficient methods. The DC electrical performance of the prepared samples was also investigated in the range of 300 to 400 K using the four-probe method. In this study, significant findings were achieved related to the functional features of the Sn_0.8_Mn_0.2_S-nanostructure-incorporated PVA films.

## 2. Methods and Materials

In this study, manganese (II) chloride tetrahydrate (MnCl_2_·4H_2_O), tin(II) chloride dihydrate (SnCl_2_·2H_2_O), and sodium sulfide nonahydrate (Na_2_S·9H_2_O), used as the source of Mn^2+^, Sn^2+^, and S^2−^ ions, respectively, were supplied by Sigma-Aldrich Co. (St. Louis, MI, USA). Polyvinyl alcohol (PVA; a degree of hydrolysis of 86–89% and molecular weight of 4 × 10^4^ g/mol.), as the host polymer, was supplied by Alfa Aesar Co. (Ward Hill, MA, USA). All reagents were used directly as supplied, with no additional purification steps. To obtain the Sn_0.8_Mn_0.2_S nanostructures, separate 0.1 M aqueous solutions of Mn^2+^, Sn^2+^, and S^2−^ ions were equipped by dissolving the appropriate precursor salts in distilled water under magnetic stirring for 30 min at room temperature. The Sn^2+^ and Mn^2+^ solutions were then combined in a 0.8:0.2 molar ratio. The S^2−^ solution was subsequently added dropwise to the Sn^2+^/Mn^2+^ mixture with continuous stirring for a further 30 min, yielding a colloidal precipitate at room temperature (~25 °C); the pH of the reacting solutions was maintained at 10. The precipitate was washed and centrifuged through three cycles. Next, the plain PVA solution was obtained by adding 1 g of PVA granules to 30 mL of double-distilled water (DDW) and stirring continuously at 70 °C for 3 h, until a clear, homogeneous solution formed. The obtained solution possessed a moderate viscosity that can be reproducibly poured and cast to produce uniform and bubble-free films. Varying weight percentages (0, 0.1, 0.5, 1.0, 5.0, and 10.0 wt%) of Sn_0.8_Mn_0.2_S nanostructures as a filler was then dispersed in the PVA solution under stirring. The filler loading (wt%) was defined as follows:

(1) $$wt\%=\;\frac{w_{filler}}{w_{filler}+w_{PVA}}\times 100\%$$

where w_filler_ and w_PVA_ are the weights of the Sn_0.8_Mn_0.2_S filler and of the PVA solution, respectively. The resulting plain and filled solutions were cast into glass Petri dishes, dried in an oven at 50 °C for 24 h, and then allowed to cool gradually over a further 24 h. The resulting films were peeled from the dishes, labeled according to the sample codes in Table 1, and stored in a desiccator containing silica-gel pouches prior to characterization. It is worth mentioning that the casting volume and Petri-dish area were kept the same for all compositions, so that the dried films had a closely comparable thickness (≈0.020–0.021 cm, as used for the optical and electrical calculations below), minimizing thickness-related bias when comparing the plain and Sn_0.8_Mn_0.2_S-loaded films. Residual moisture retained during the drying protocol can also influence the free volume and optical/electrical response of PVA-based coatings [31,32].

The structural properties of the plain and filled films were recorded over 2θ range from 10° to 80° with 0.02° step using an X-ray diffraction (XRD, Bruker, Billerica, MA, USA; D8-Advanced). It functioned using Cu K_α_ (1.5400598 Å), voltage of a 40 kVolt, and 40 mA. The chemical bonding and bond vibration were examined by Fourier-transform infrared spectroscopy (FTIR, Shimadzu, Kyoto, Japan), with transmittance spectra recorded over the 500–4000 cm^−1^ range. The morphology of the Sn_0.8_Mn_0.2_S nanostructures was studied using a JEOL scanning electron microscope (SEM) (JEOL, Tokyo, Japan). The optical properties (transmittance and absorbance) of the films were measured using a JASCO V670 UV–Vis–NIR spectrophotometer (JASCO, Tokyo, Japan). Based on the absorbance and transmittance data, the optical parameters absorption coefficient (α), optical energy gap (E_g_), refractive index (n), dielectric constants (real *ε_r_* and imaginary *ε_i_* parts), extinction coefficient (K), and optical conductivity (*σ_opt_*_._) were obtained from [33,34,35,36]:

(2) $$\alpha =\;\frac{2.303*Abs.}{d}$$

(3) $$\alpha h\nu ={A(h\nu -E_{g})}^{m}$$

(4) $$K=\frac{\alpha \lambda }{4\pi }$$

(5) $$\frac{n^{2}-1}{n^{2}+1}=1-\sqrt{E_{g}/20}$$

(6) $$\varepsilon_{r}=n^{2}-K^{2}$$

(7) $$\varepsilon_{i}=2\;nK$$

(8) $$\sigma_{opt.}=\frac{\alpha nC}{4\pi }$$

where *d* is the thickness, *hν* is the incident photons energy, *A* is a constant, *m* is a parameter controlled by the kind of the material’s electronic transitions, and C is the speed of light. It should be noted that Equation (5) is an approximate empirical relation that returns a single, wavelength-independent refractive index value from the optical bandgap, rather than the full dispersion n(λ). The DC electrical resistance (R) and conductivity of the plain and filled films were measured using a four-probe (Keithley) system over the temperature range 300 to 400 K, with a heating rate of 1 K.min^−1^. The samples were disk-shaped with an area of 0.58 cm^2^ and a thickness of 0.021 cm for the electrical investigations. The activation energy (E_ac_) for all samples was determined using the Arrhenius equation as [37,38,39]:

(9) $$\sigma_{dc}=\;\sigma_{o}e^{-\frac{E_{ac}}{k_{B}T}}$$

where k_B_ (=8.26 × 10^−5^ eV/K [40]) and σ_o_ are the Boltzmann constant and the pre-exponential factor, respectively.

## 3. Results and Discussion

### 3.1. FTIR Analysis

**Figure 1:**
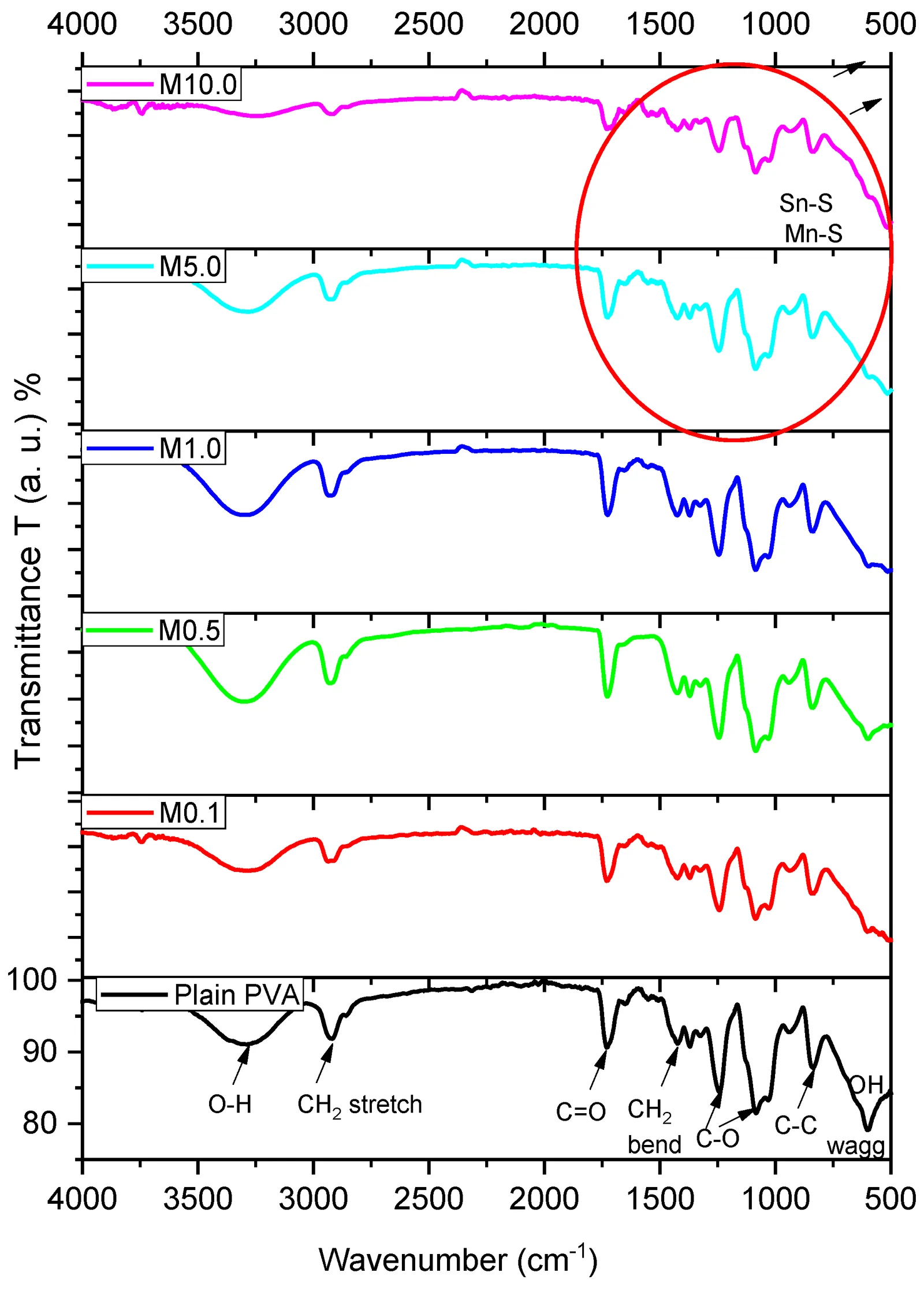
FTIR transmittance spectra of the plain and Sn_0.8_Mn_0.2_S/PVA films (M0.1 to M10.0).

The FTIR transmittance spectra of the plain and Sn_0.8_Mn_0.2_S-nanostructure-incorporated PVA films with various filler loadings are presented in Figure 1 across the region from 500 cm^−1^ to 4000 cm^−1^. The spectra of the plain PVA film and the lower-loading films (M0.1, M0.5, and M1.0) show a series of well-resolved transmittance minima characteristics of PVA. A broad absorption band near 3296 cm^−1^ is due to O-H stretching groups of adsorbed water [9]. The band centered at 2926 cm^−1^ refers to the C-H stretching of the CH_2_ backbone [41]. The bands between approximately 1800 and 750 cm^−1^ are generally attributed to the C=O/residual-acetate absorption (~1726 cm^−1^) [41,42], CH_2_ bending (~1430 cm^−1^) [9], OH rocking/CH wagging (~1325 cm^−1^) [9], C-O stretching of the crystalline and amorphous PVA sequences (~1243 cm^−1^ and ~1082 cm^−1^, respectively), CH_2_ rocking (~934 cm^−1^), C-C skeletal stretching (~838 cm^−1^), and OH wagging (~600 cm^−1^) consistent with the well-established PVA band [9,43,44]. As the loading of Sn_0.8_Mn_0.2_S is increased to 5 wt.% and 10 wt.%, the fine structure in both of the emphasized spectral windows of Figure 1 (the ~1750–1450 cm^−1^ window where the C=O and CH_2_-bending bands lie, and the ~850–650 cm^−1^ window where the C-C skeletal band can be found) shows progressive broadening, together with partial overlaps and the slight shifting of these absorption bands indicating new hydrogen-bonding/coordination interactions between the hydroxyl and carbonyl groups of PVA and the incorporated Sn_0.8_Mn_0.2_S nanostructures. Consequently, the structure of the PVA host is effectively reformed with the filling of Sn_0.8_Mn_0.2_S nanostructures, which is compatible with the literature [1,3,10,43].

### 3.2. Structural (XRD) Analysis

**Figure 2:**
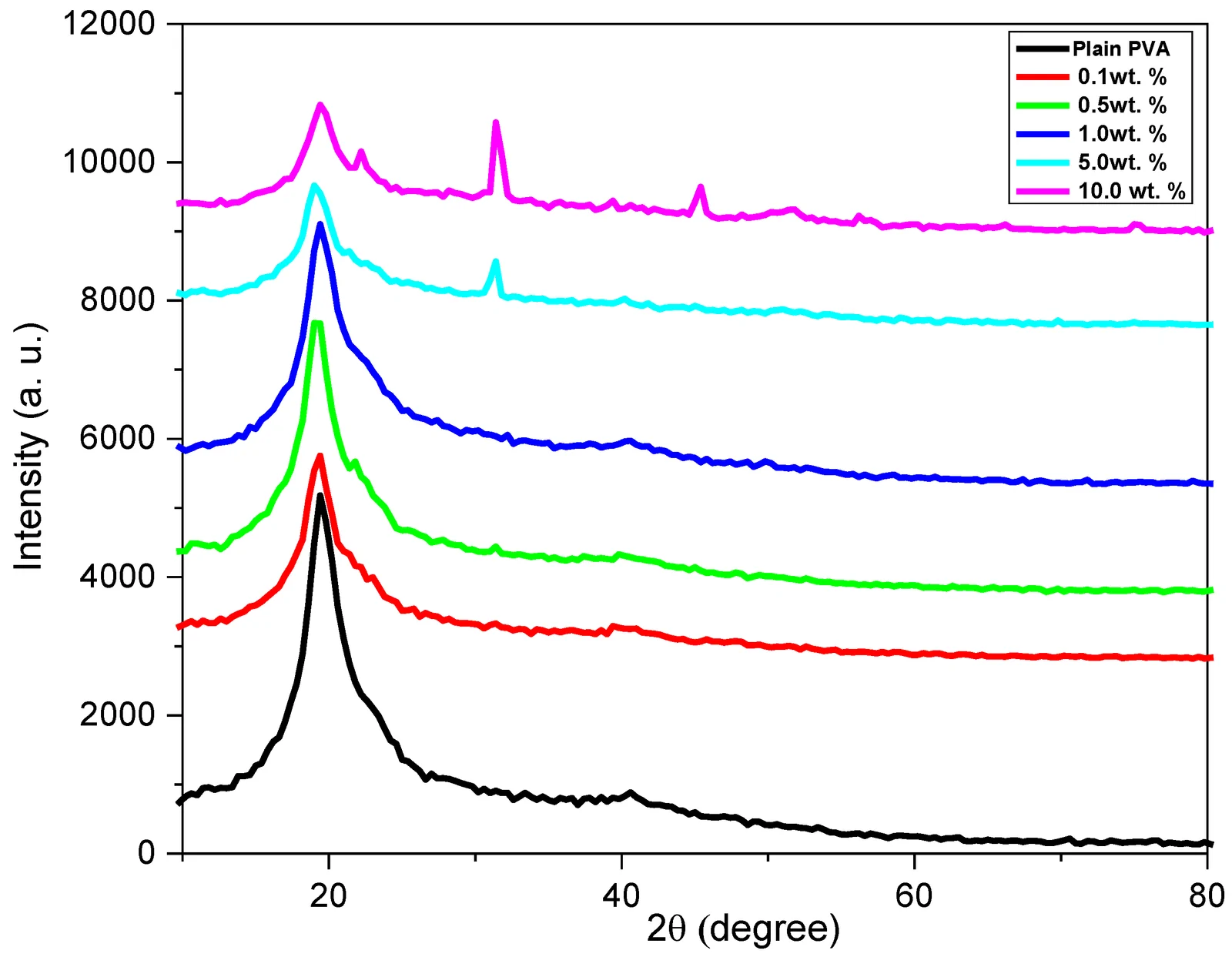
XRD of the plain and Sn_0.8_Mn_0.2_S/PVA nanocomposite films (M0.1–M10.0).

The structural properties of the plain and Sn_0.8_Mn_0.2_S nanocomposite films were investigated on the basis of X-ray measurements. Figure 2 illustrates the XRD patterns of all samples (plain PVA to M10.0) in the range of 2θ = 20–80°. The diffraction pattern of the plain PVA film confirms the typical diffraction peak at 2θ = 19.60°, which is also observed in previous reports of semi-crystalline PVA [45]. Together with the expected semicrystalline nature of the host polymer before the filling, this reflection, combined with the amorphous background that is generally seen in PVA-based films, confirms the expected situation. The intensity of this broad peak decreased due to Sn_0.8_Mn_0.2_S incorporation, indicating to a decrease in the crystalline feature of the PVA medium. The variation in the XRD measurement of the PVA reveals the interaction between the Sn_0.8_Mn_0.2_S nanostructures and the backbones of the PVA molecules. These investigations are consistent with previous work related to Co_3_O_4_-NP-filled PVA–PVP films [46]. The XRD peaks reported in the high concentrations of Sn_0.8_Mn_0.2_S nanostructure films (M5.0 and M10.0) correspond to the XRD reflections of the Sn_0.8_Mn_0.2_S nanostructures (JCPDF 73–1859 [47]) and are well-matched with those reported in Refs. [48,49].

### 3.3. Morphological (SEM) Analysis

**Figure 3:**
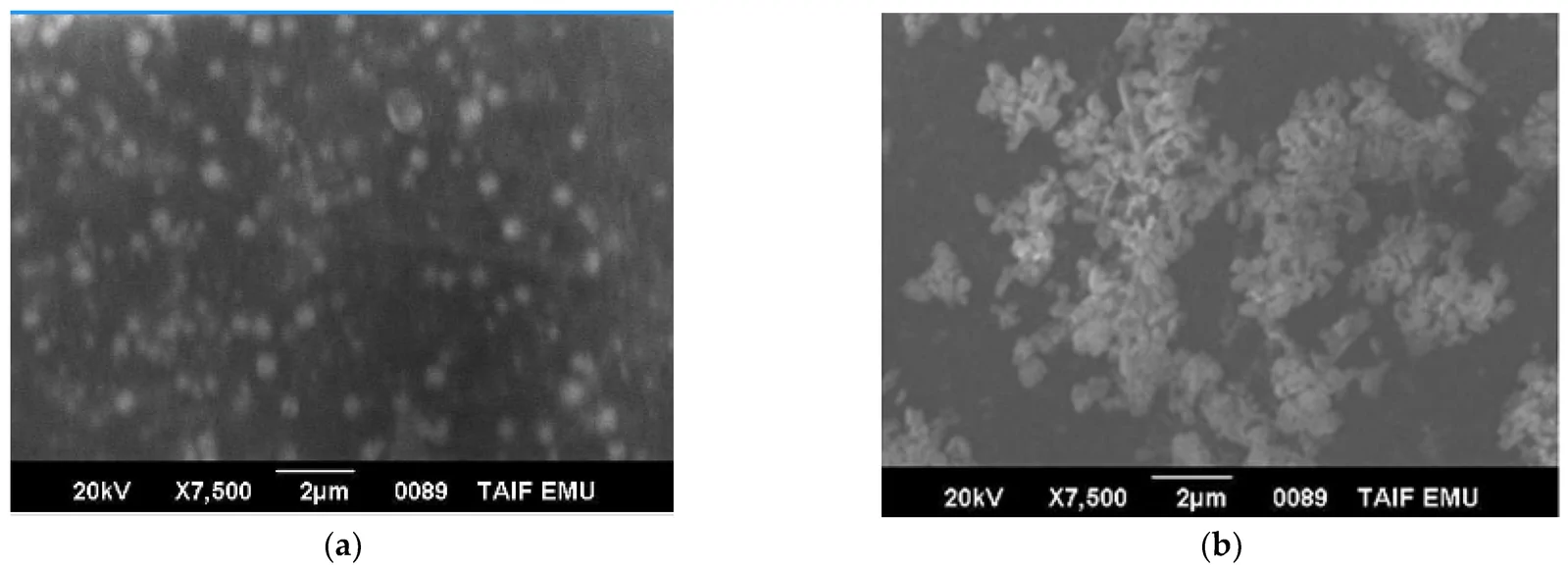
SEM micrographs of (**a**) 1.0 wt.% and (**b**) 10.0 wt.% of Sn_0.8_Mn_0.2_S-nanostructure-incorporated PVA film.

The scanning electron microscopy (SEM) technique was applied to explore the surface morphology of Sn_0.8_Mn_0.2_S-nanostructure-incorporated PVA film at 20 kV and ×7500 magnification (Figure 3). Figure 3a,b display the SEM micrographs of low (M1.0) and high (M10.0) Sn_0.8_Mn_0.2_S nanostructure concentrations, respectively. It is clear that the Sn_0.8_Mn_0.2_S nanostructures are distributed homogeneously within the PVA medium for low amounts, while the SEM micrograph of a high Sn_0.8_Mn_0.2_S nanostructure amount is flake-/petal-like with heavy agglomeration, instead of being individually dispersed. This type of agglomeration is often observed in the nanostructured metal sulfides in which the precipitations were carried out in aqueous solution without any other surfactant or dispersion step, where the nanoparticles tend to reduce their surface energy by aggregation. As reported in other PVA nanocomposites [50], such as that related to SnO NPs, the same trend of homogeneous dispersion to visible aggregation has been noticed with homogeneous dispersion of up to 1 wt.% loading, while aggregation and cluster formation were visible for higher concentrations. The agglomeration of the filler can locally enrich the Sn_0.8_Mn_0.2_S phase. The morphological investigations are consistent with the above XRD analysis. The SEM findings point to the basis for distinguishing the two proposed electrical mechanisms (Section 3.5). The homogeneous dispersion observed in low loadings up to 1.0 wt.% supports continuous, low-resistance hopping pathways as the dominant mechanism in that range, whereas the flake-like agglomeration visible at 5.0 and 10.0 wt.% is the structural correlate of the disrupted conductive network and the additional charge-trapping/vacancy-related centers invoked to explain the partial recovery of resistivity and activation energy at the highest loadings.

### 3.4. Analysis of Optical Properties

**Figure 4:**
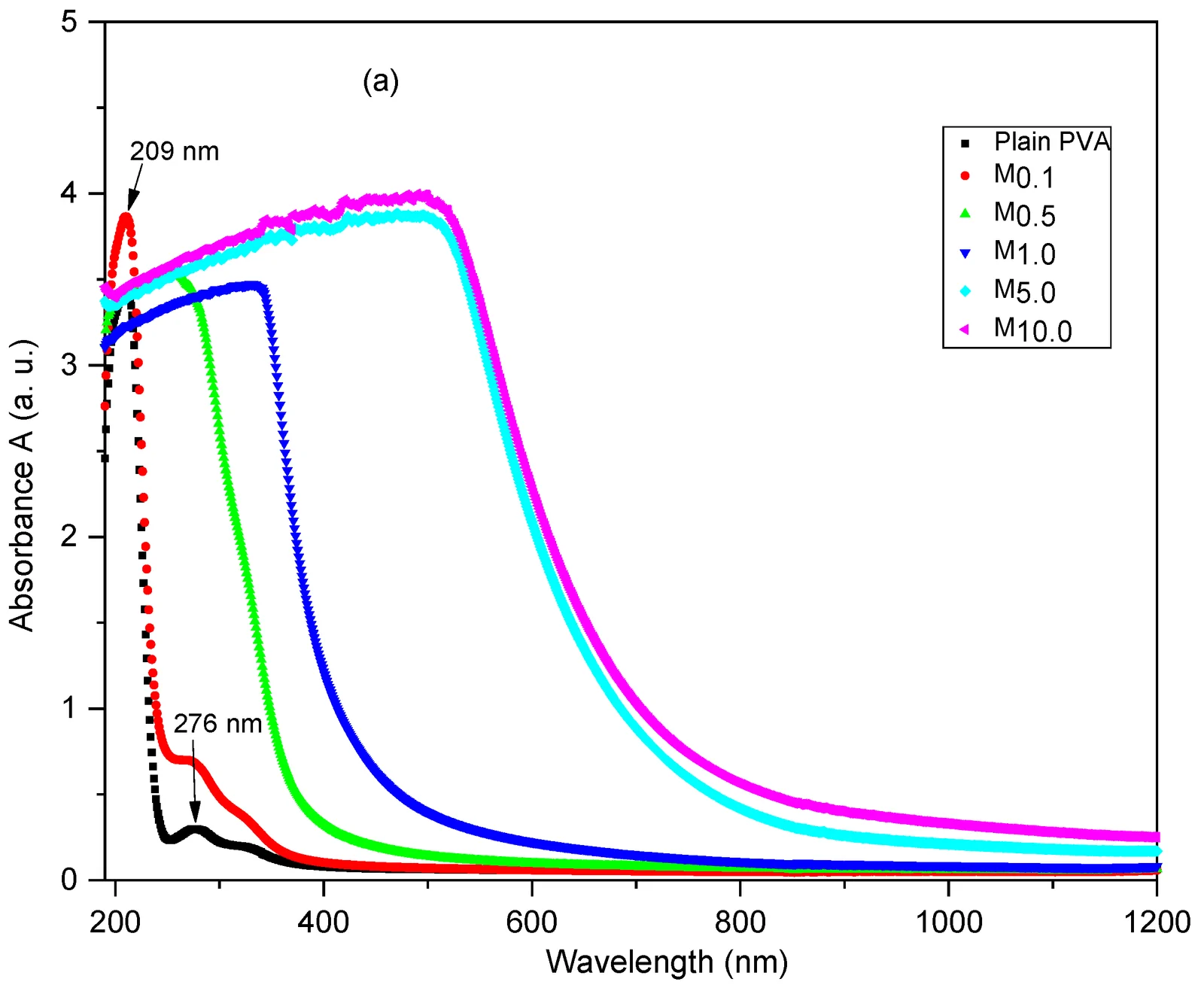

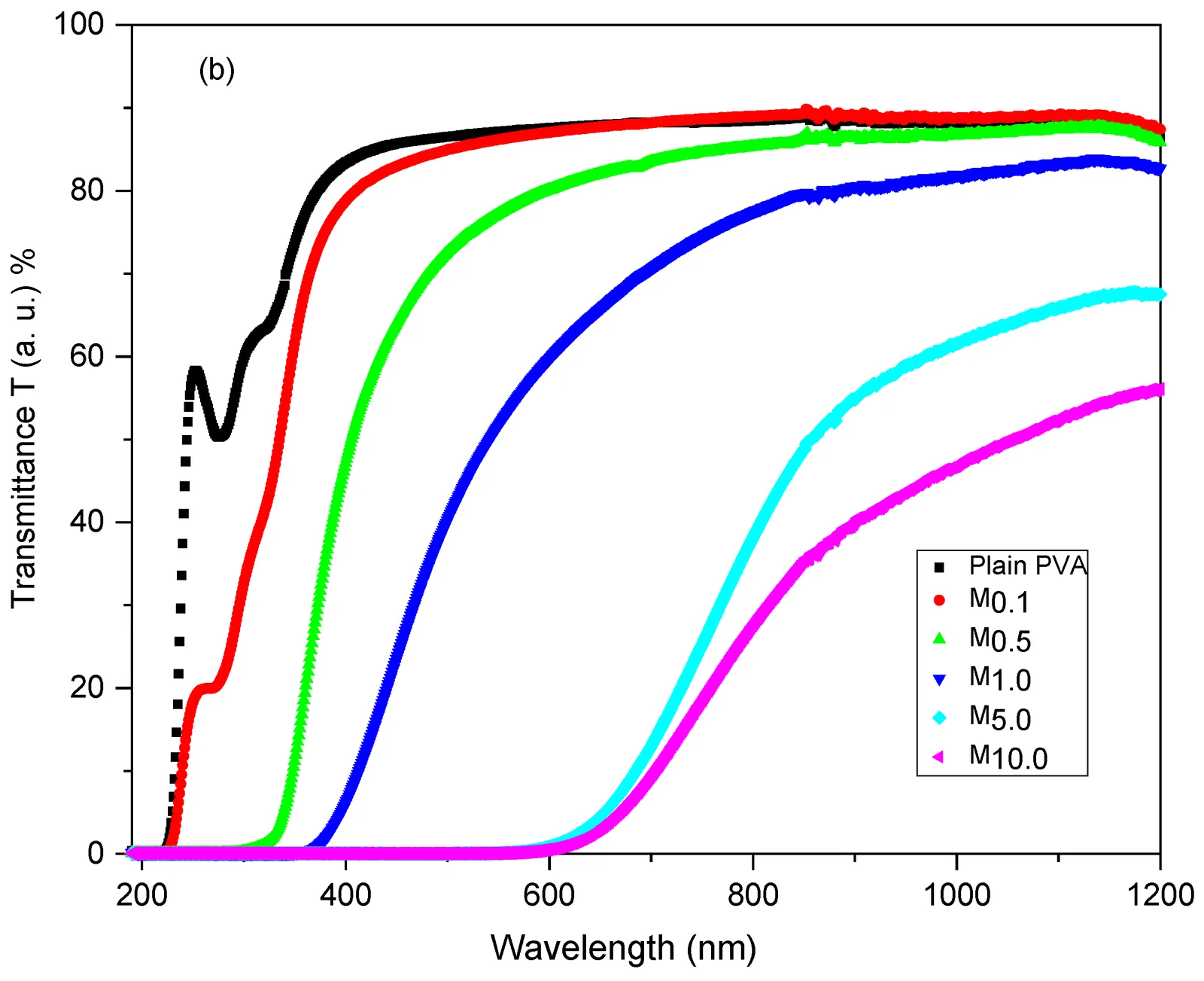
UV–Visible–NIR absorption (**a**,**b**) transmittance of the plain and Sn_0.8_Mn_0.2_S/PVA nanocomposite films (M0.1–M10.0).

The UV–Visible and near-infrared (UV–Vis–NIR) absorption and transmittance spectra of all Sn_0.8_Mn_0.2_S films were measured and compared to those of the plain PVA film as a function of the amount of Sn_0.8_Mn_0.2_S nanostructures. Figure 4a reveals that the absorption spectrum of the plain PVA film has two major absorption peaks at 209 nm and 276 nm, both corresponding to π→π* electronic transitions of the PVA chain, while the absorption edge at around 326 nm is attributed to the n→π* inter-band transition [3]. The transmittance of the Sn_0.8_Mn_0.2_S-nanostructure-incorporated PVA films is lower than that of the plain PVA film in both visible and near-infrared ranges and lower as the amount of filler increases, as illustrated in Figure 4b. The transmittance of the plain PVA films is decreased upon the incorporation of Sn_0.8_Mn_0.2_S nanostructures. The filled films also show a clear red-shift for the absorption edges compared with the plain PVA film (Figure 4a). The cut-off edge of the PVA film is shifted from 221 nm to 619 nm for the 10 wt.% of Sn_0.8_Mn_0.2_S nanostructure loading. This behavior designates a clear decrease in the optical energy gap with an increasing Sn_0.8_Mn_0.2_S nanostructure content. The observed variations demonstrate the possible tunability of the transmittance of the PVA film with Sn_0.8_Mn_0.2_S nanostructure loading content, which is directly applicable to optoelectronic applications where a required optical response is needed.

**Figure 5:**
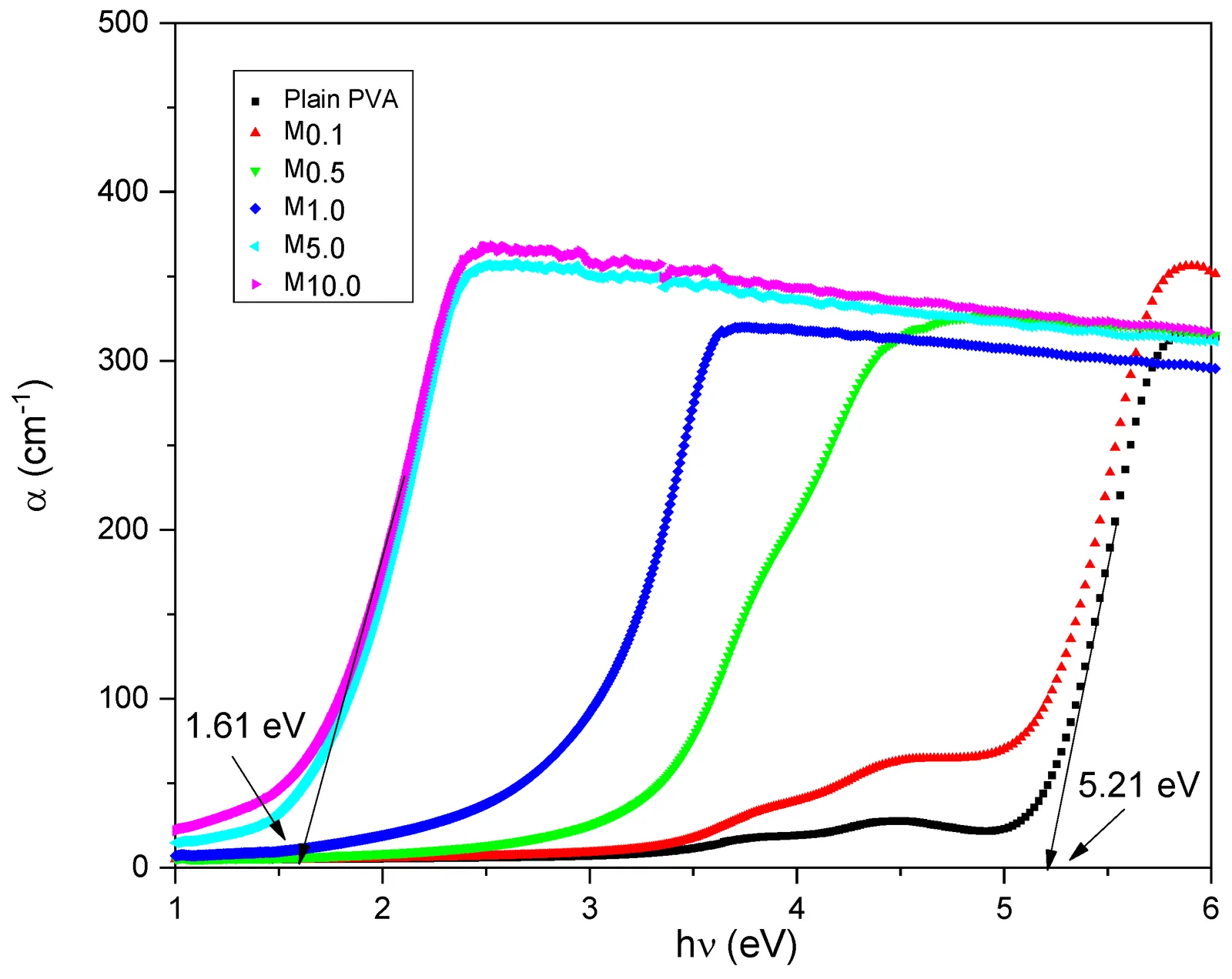
Optical absorption coefficient of Sn_0.8_Mn_0.2_S/PVA nanocomposite films (M0.1–M10.0).

**Figure 6:**
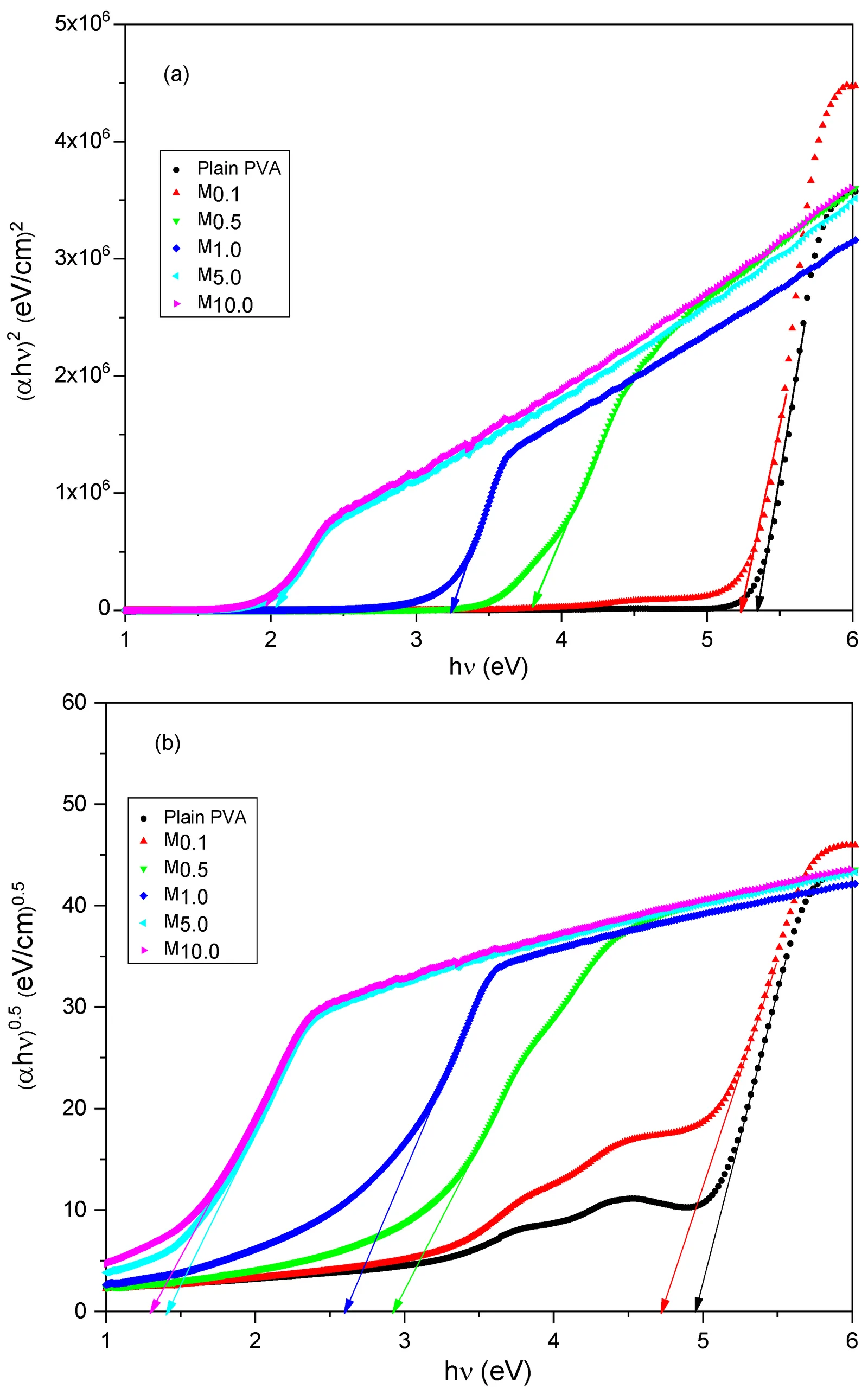
Tauc’s plots (**a**) direct and (**b**) indirect electronic transition of Sn_0.8_Mn_0.2_S/PVA nanocomposite films (M0.1–M10.0).

**Figure 7:**
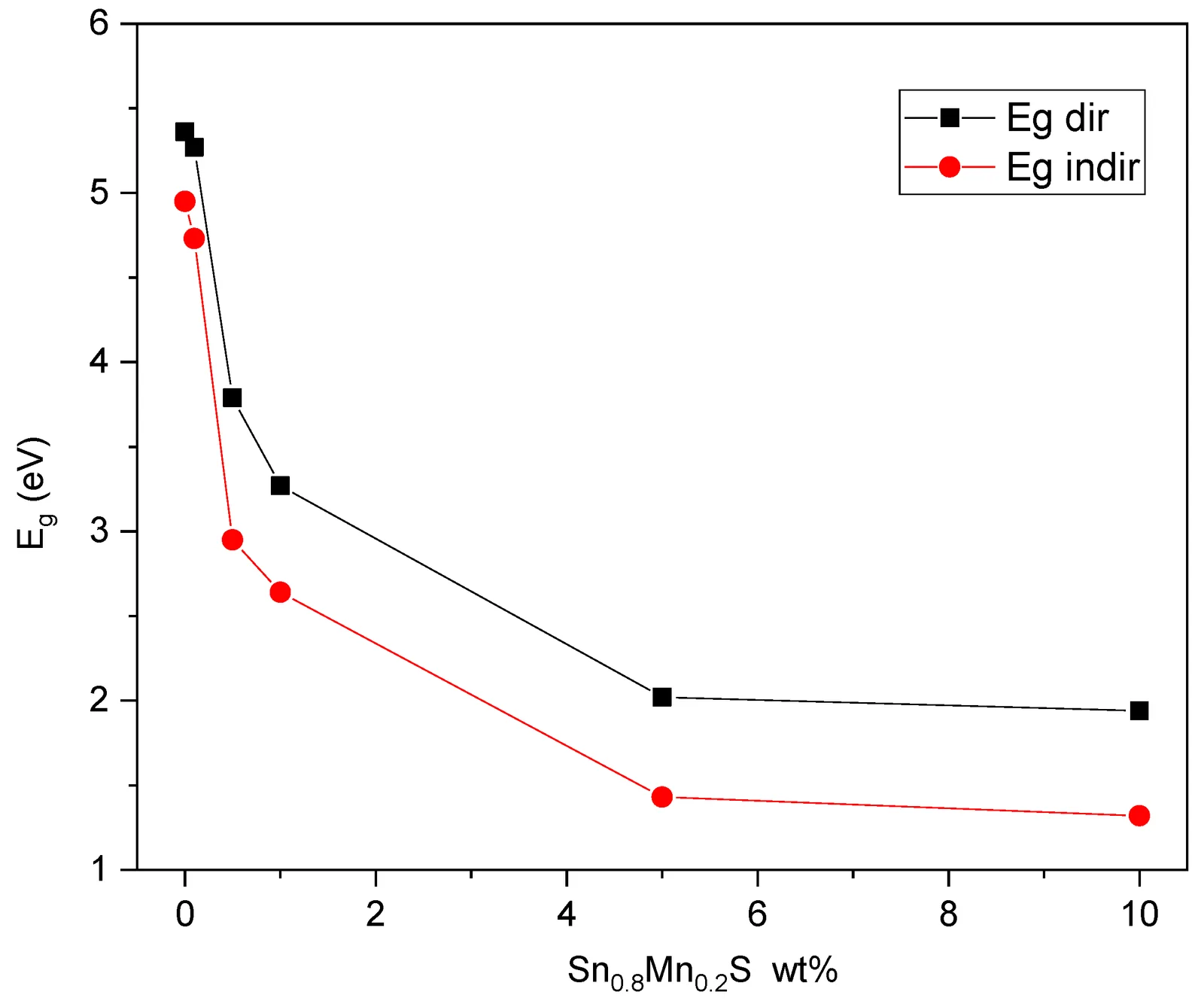
Optical bandgap performance of Sn_0.8_Mn_0.2_S/PVA nanocomposite films (M0.1–M10.0).

The optical absorption coefficient (α) was determined by Equation (2), and it increases with increasing Sn_0.8_Mn_0.2_S content in the range 0.1 to 10 wt.%, as displayed in Figure 5. The α performance is satisfactory as a result of the larger dispersion of the absorbance due to the Sn_0.8_Mn_0.2_S nanostructures. The optical band gap of all samples could be estimated by extrapolating the linear portions of α plots to the photon energy axis. It is found that the optical band gap varies from 5.21 eV for the plain PVA to 1.61 eV for the 10 wt.% of Sn_0.8_Mn_0.2_S incorporation. The determination of the optical band gap is also confirmed by applying Tauc’s method, as shown in Equation (3). The direct/indirect optical energy gap of all films was deduced via the plotting of (αhν)^2^ and (αhν)^0.5^ versus hν as shown in Figure 6. The intercept of the extrapolated linear part of each film curve with the hν-axis is the optical band gap (E_g_) value. The resulting E_g_ through Tauc’s method is presented in Figure 7. As the content of Sn_0.8_Mn_0.2_S nanostructures increases, both the direct and indirect optical band gap decrease significantly from 5.36 eV and 4.95 eV for the plain PVA film to 1.94 eV and 1.32 eV for the 10.0 wt.% film, respectively. The direct optical transition values are systematically higher than the corresponding indirect ones for every composition studied. This is consistent with a direct, momentum-conserving transition probing closer to the true fundamental gap of the disordered PVA/Sn0.8Mn0.2S network, whereas the indirect, phonon-assisted fit instead captures a lower-energy absorption channel mediated by the amorphous PVA matrix and by the localized sub-gap states introduced by the filler. It is noticed that the optical bandgap for the plain PVA film is larger than for any Sn_0.8_Mn_0.2_S incorporated PVA film. In terms of the intrinsic bandgaps of the binary components, the bandgap of PVA (~5.4 eV) compared to those of MnS (~3.7 eV) and SnS (~1.3 eV) can be rationalized for the reduction in the optical bandgap with increasing Sn_0.8_Mn_0.2_S content. The introduction of localized sub-gap states within the PVA bandgap is expected to be the reason for both the red-shift of the absorption edge and the narrowing of E_g_ with the incorporation of Sn_0.8_Mn_0.2_S nanostructures. Physically, this is attributed to hybridized states derived from the Sn 5s/5p and Mn 3d orbitals overlapping with the S 3p states, which introduce localized levels close to the valence and conduction band edges of the host PVA and thereby narrow the effective optical gap as the Sn_0.8_Mn_0.2_S content increases. In combination with the clearly observed decrease in transmittance with increasing loadings, the results presented here show that the optical response of PVA can be varied over a broad range from a plain PVA with a large gap and high transmittance, up to a comparatively small gap with a high absorption of the nanocomposite, just by varying the Sn_0.8_Mn_0.2_S loading. The tunability of the material opens up the possibility of using Sn_0.8_Mn_0.2_S/PVA nanocomposite films in optoelectronic devices for which it is necessary to achieve a specific optical response. It is worth noting that the bandgap reduction here is much larger than previously reported for PVA with wide-gap oxide nanofillers [3], and more comparable to the results obtained with narrow-gap sulfide nanofillers. For instance, a small reduction in the optical bandgap from 4.59 to 4.18 eV was observed upon the incorporation of SnO nanoparticles into PVA up to loadings of 5 wt.% [50], while the incorporation of Cu/MnS nanostructures into the PVA/PVP blend reported by Heiba et al. [51] results in a significant and concentration-dependent bandgap reduction with the changing from an indirect-allowed to direct-allowed optical transition character. In the present Sn_0.8_Mn_0.2_S/PVA system, the reduction of a similar order of magnitude (from 5.40 to 1.95 eV) is consistent with the general expectation that narrow-gap metal-sulfide fillers like MnS and SnS-based nanostructures are much more effective than wide-gap oxide fillers in introducing the sub-gap states responsible for a large reduction in the bandgap of PVA. This is consistent with the composition-dependent bandgap narrowing of the PVA film via incorporating Sn_0.8_Mn_0.2_S nanostructures, highlighting the sensitivity of the structure of the PVA polymer, and, thus, its optical response.

**Figure 8:**
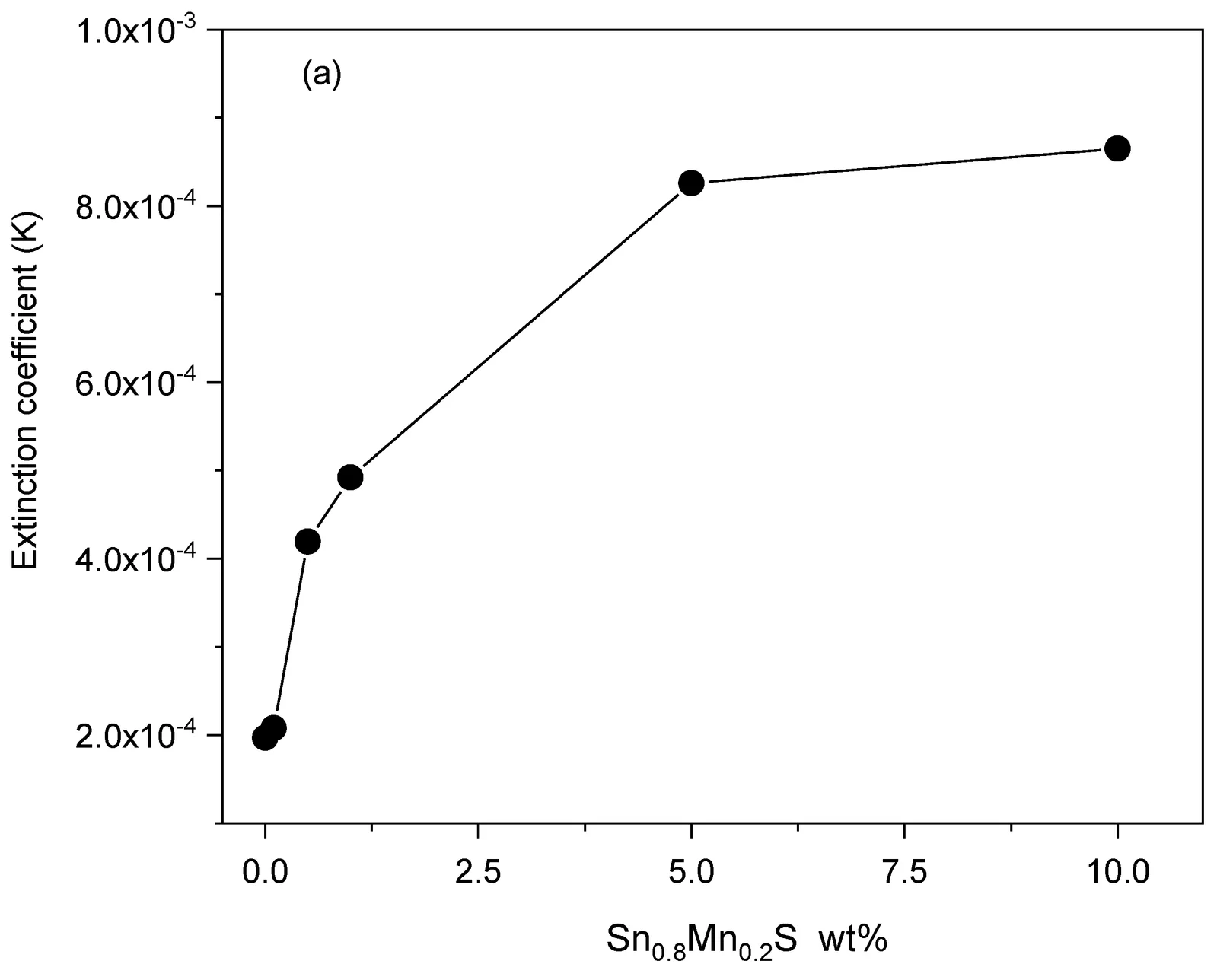

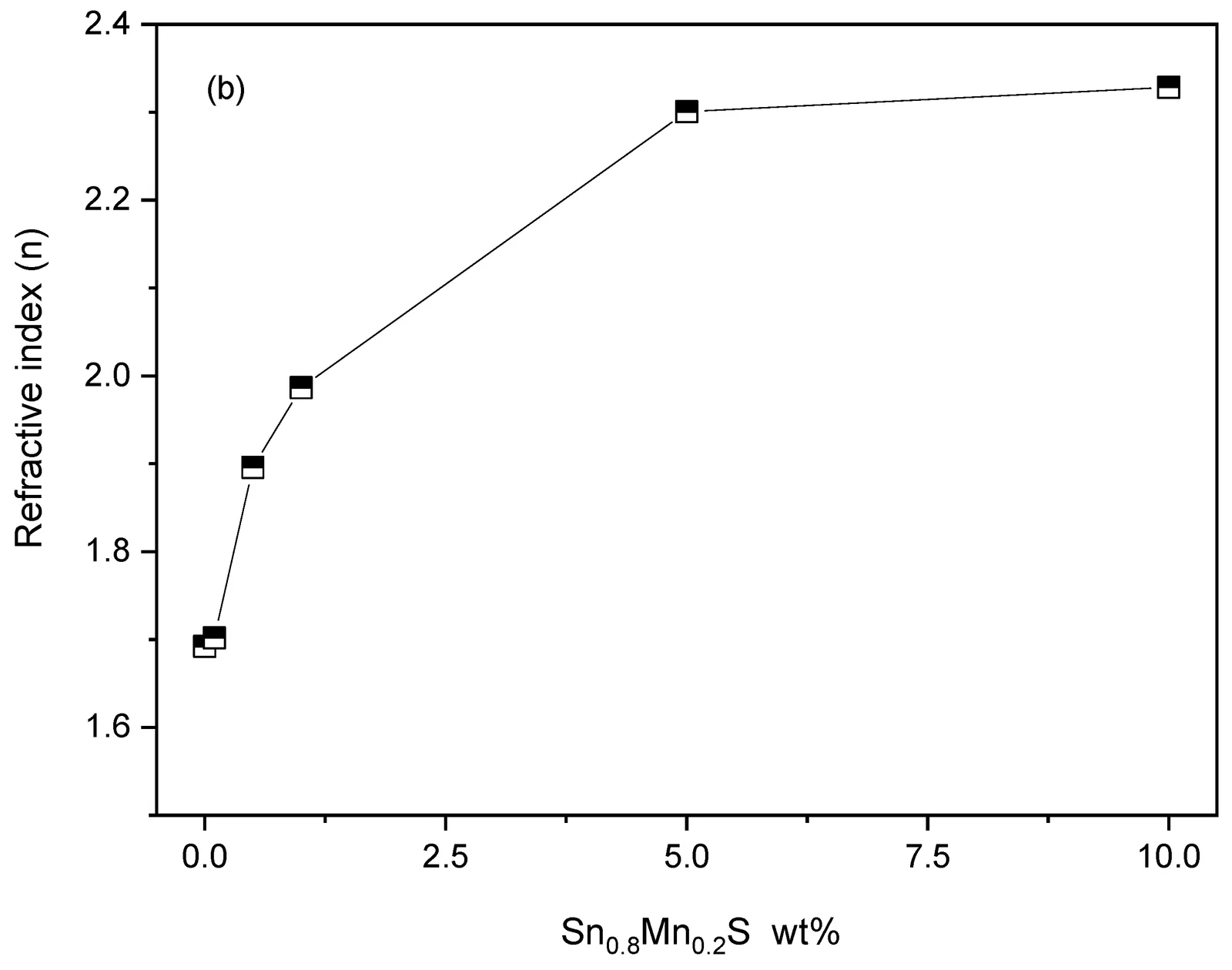
(**a**) Extinction coefficient (K) and (**b**) refractive index (n) performance of Sn_0.8_Mn_0.2_S/PVA nanocomposite films (M0.1–M10.0).

In addition to the optical bandgap, the refractive index (n), extinction coefficient (K), dielectric constant, and optical conductivity (σ_opt._) are vital optical parameters to be considered for determining what material can be used for such an optoelectronic application. These parameters significantly influence the light propagation, reflection losses, and the behavior of photogenerated carriers within films. The extinction coefficient (K) is related to the optical absorption coefficient (Equation (4)) in the strong-absorption region above the absorption edge. The performance of all samples’ refractive index (n) is determined by the Dimitrov–Sakka formula (Equation (5)) [36]. The obtained data of both the extinction coefficient (K) and refractive index (n) for all samples (M0.1–M10.0) are shown in Figure 8a and Figure 8b, respectively. It is noted that the K value rises from 1.97084 × 10^−4^ for the plain PVA film to 8.65126 × 10^−4^ due to 10 wt.% of Sn_0.8_Mn_0.2_S incorporation, while the refractive index n of the PVA film is increased from 1.69 to 2.33, highlighting the prepared samples for a lot of optical applications. These results are mainly attributed to the enhancement in the absorption of the PVA as a result of Sn_0.8_Mn_0.2_S loading. Similarly, Ragab et al. [16] showed that MWCNT–graphene loading enhanced the refractive index of the PVA–PEO blend.

**Figure 9:**
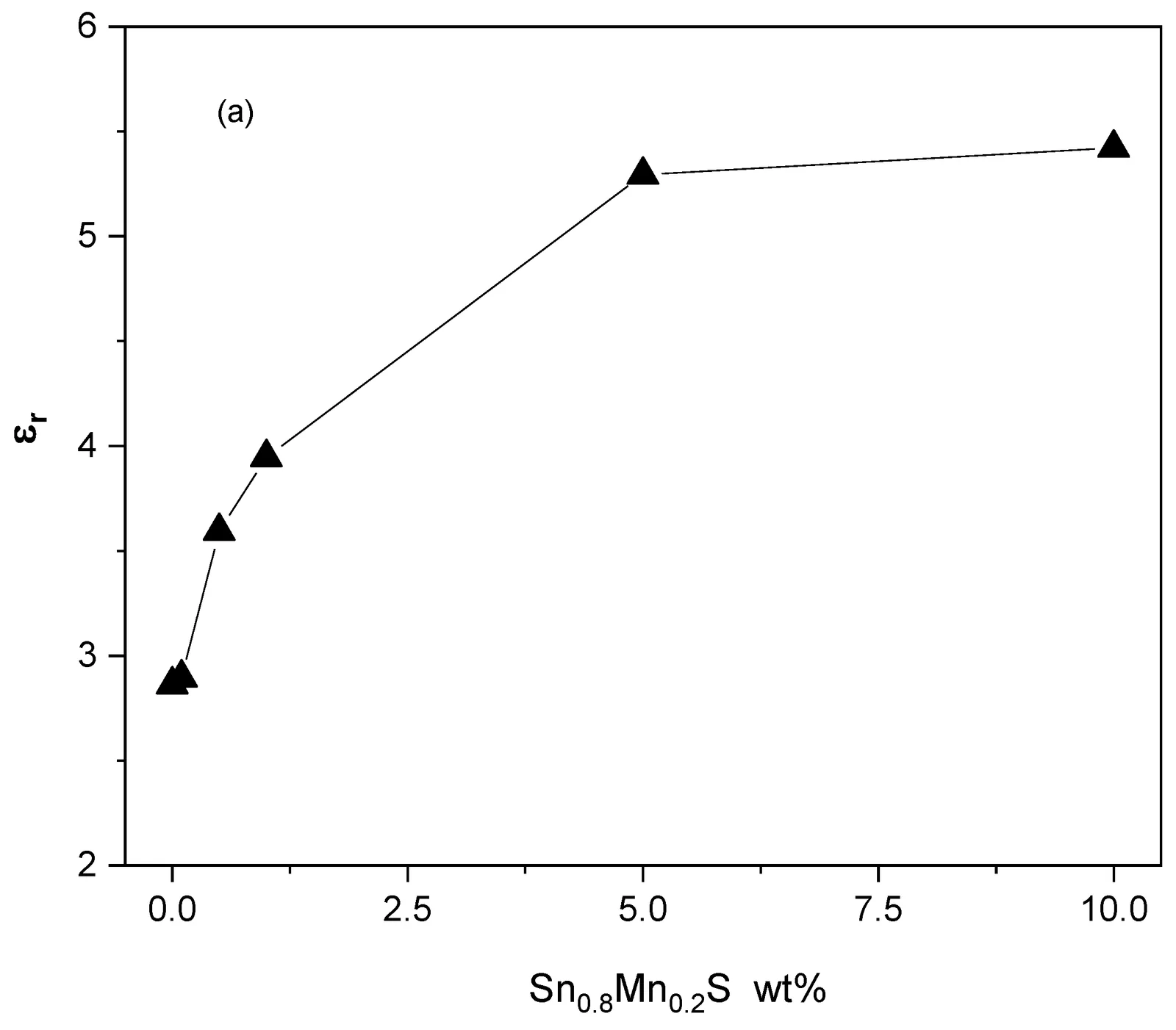

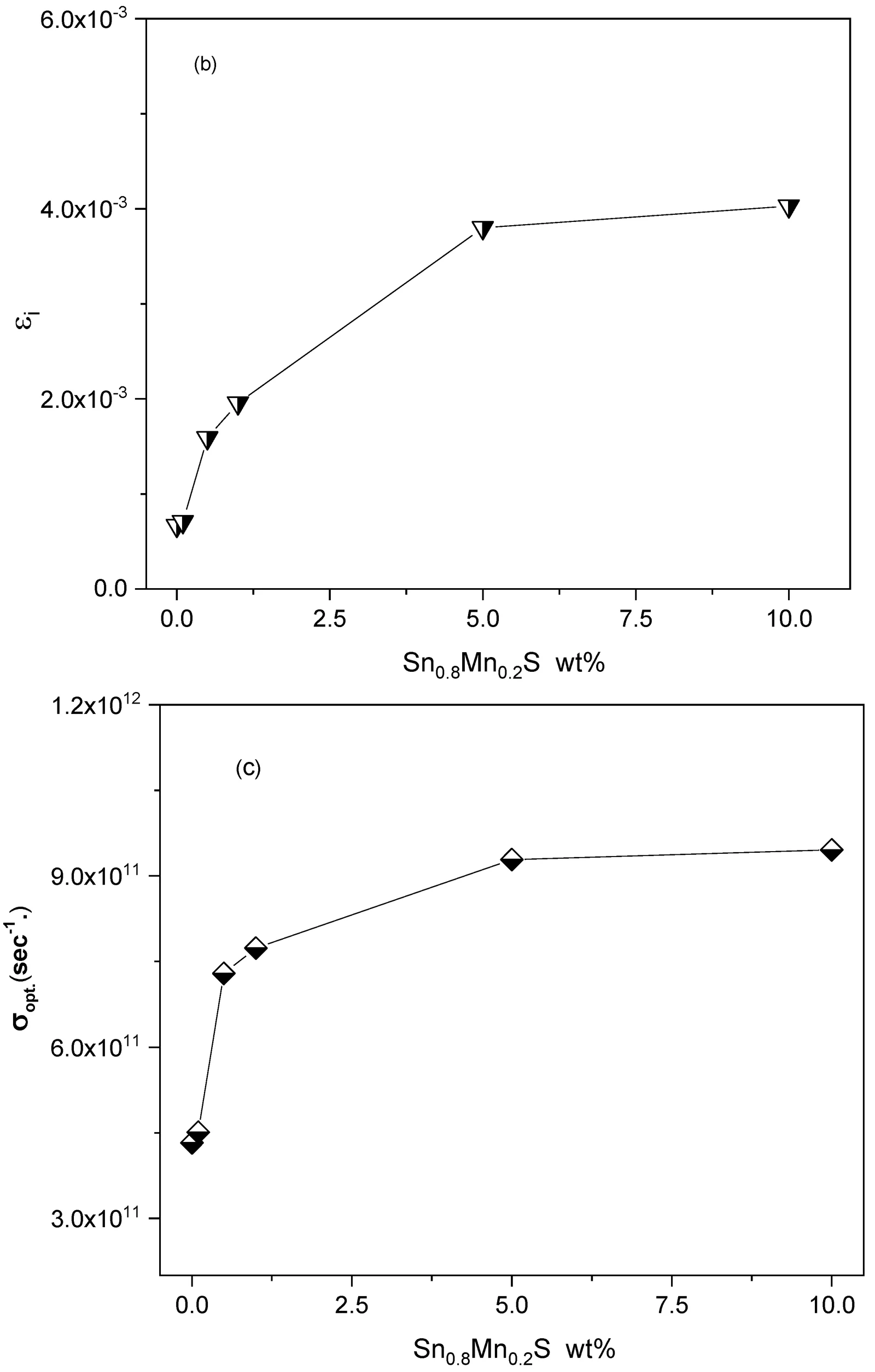
(**a**) Real, (**b**) imaginary dielectric, and (**c**) optical conductivity of Sn_0.8_Mn_0.2_S/PVA nanocomposite films (M0.1–M10.0).

Moreover, the dielectric constant and optical conductivity (σ_opt._) are all related to the index of refraction and extinction coefficients as shown in Equations (6)–(8). The performance of the real part of the dielectric constant (ε_r_), imaginary part (ε_i_), and optical conductivity (σ_opt._) for all samples are presented in Figure 9a, Figure 9b and Figure 9c, respectively. A significant enhancement in all PVAs’ optical constants is achieved through Sn_0.8_Mn_0.2_S nanostructure incorporation. The real dielectric constant rose from 2.86 to 5.42, the imaginary part increased from 6.7 × 10^−4^ to 4.0 × 10^−3^, and the optical conductivity increased from 4.33 × 10^11^ s^−1^ to 9.46 × 10^11^ s^−1^ upon 10 wt.% of Sn_0.8_Mn_0.2_S loading. The enhancement in the dielectric constants of the PVA film as well as the optical conductivity through Sn_0.8_Mn_0.2_S incorporation highlights the samples’ potential uses for energy storage and supercapacitor fabrication.

### 3.5. Electrical Properties Analysis

**Figure 10:**
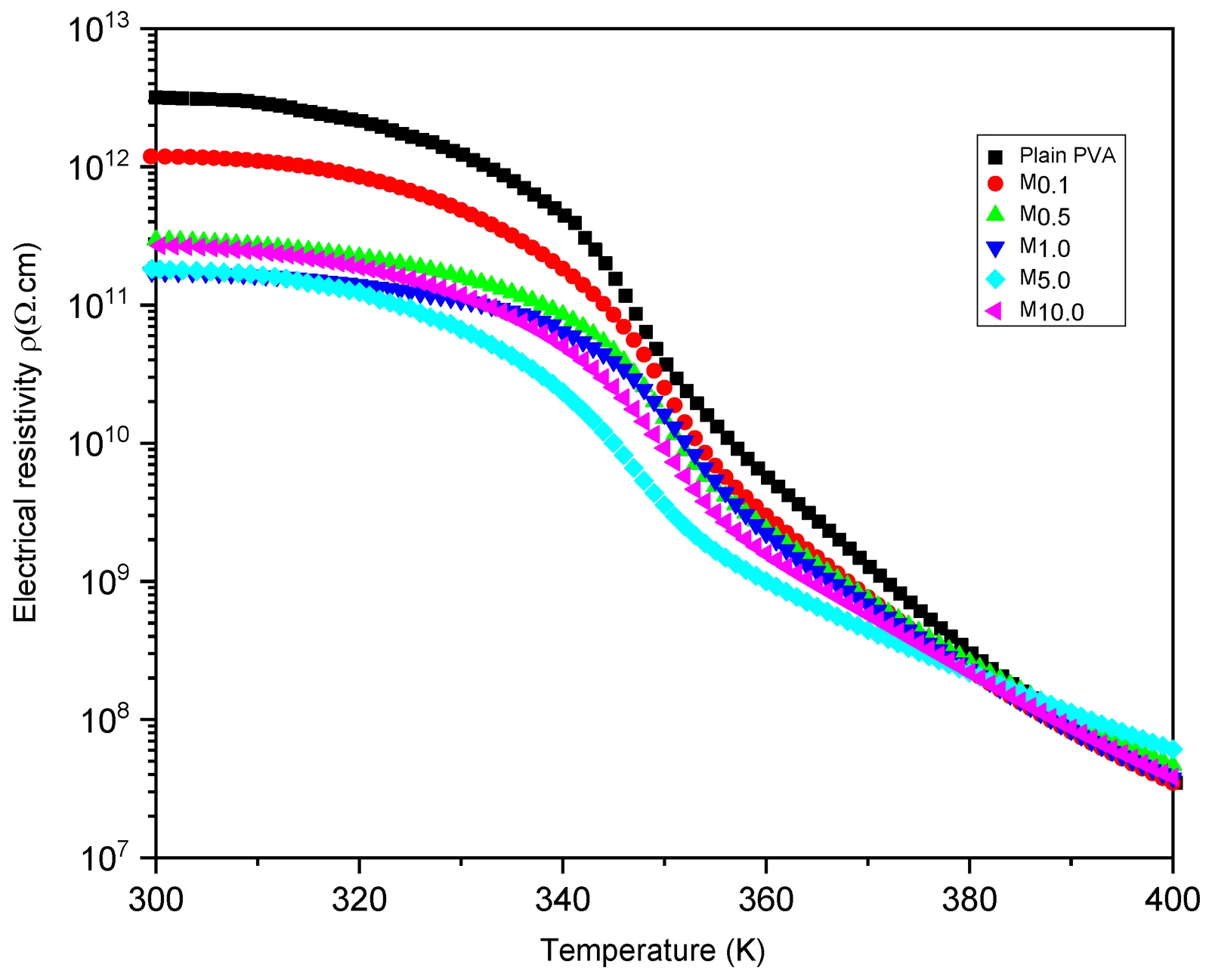
Electrical resistivity of Sn_0.8_Mn_0.2_S/PVA nanocomposite films (M0.1–M10.0).

The electrical resistance (R) of the plain PVA film and different Sn_0.8_Mn_0.2_S/PVA (0.1–10 wt%) nanocomposite films were measured by a four-probe measurement system (Keithley). The electrical measurements were made as the temperature was ramped up from 300 K to 400 K at a controlled rate of 1 K/min. Figure 10 displays the DC-electrical resistivity (ρ) of all samples. It is clear that the DC electrical resistivity decreased with increasing the Sn_0.8_Mn_0.2_S amount. Moreover, the increase in the temperature causes a decrease in the electrical resistivity ρ. The opposite trend is observed for the electrical conductivity (σ = 1/ρ [13,52]) for films. For example, at 300 K, the resistivity of PVA decreased from 3.2 × 10^12^ Ω.cm to 1.74 × 10^11^ Ω.cm upon 1 wt.% of Sn_0.8_Mn_0.2_S nanostructure loading, while the ρ value returned to rise again for both 5% and 10% of Sn_0.8_Mn_0.2_S loading. The decrease in the electrical resistivity is mainly attributed to created networks that formed due to Sn_0.8_Mn_0.2_S nanostructure loading. The loaded nanostructures facilitate the motion and hopping of the charge carriers and, hence, significantly improve the electrical conductivity of the whole medium, while the extra loadings may form trapping electron–hole pair centers that cause a reduction in the electrical conductivity. Moreover, the created vacancies within the nanocomposite medium play a dominant role for high Sn_0.8_Mn_0.2_S loadings. It is worth noting that the electrical measurements investigated in this study were carried out between 300 and 400 K, which is below the thermal-decomposition onset reported for PVA (typically above~200 °C [53]).

**Figure 11:**
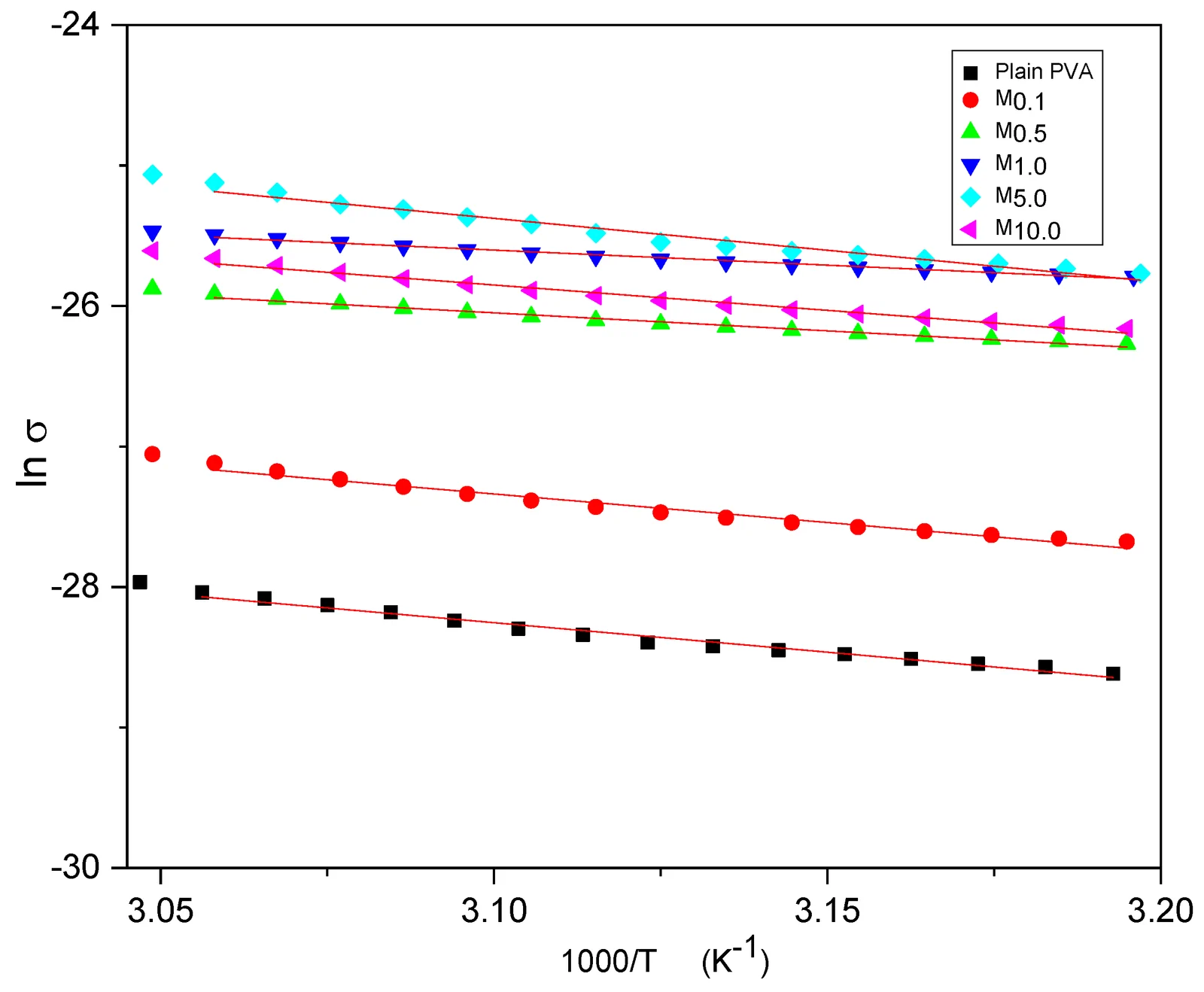
Arrhenius plot of Sn_0.8_Mn_0.2_S/PVA nanocomposite films (M0.1–M10.0).

Moreover, the activation energy (E_ac_) of the plain PVA and different Sn_0.8_Mn_0.2_S/PVA nanocomposite films was investigated using the Arrhenius relation (Equation (9)) by plotting ln σ_dc_ versus 1000/T as illustrated in Figure 11. The activation energy E_ac_ presents information about the created charge carriers within the nanocomposite medium [54]. The found E_ac_ values are listed in Table 2. According to Figure 11, one can conclude that the prepared nanocomposite films follow the Arrhenius theory as described in Refs. [55,56,57]. The obtained E_ac_ value of the plain PVA film is 0.36 eV, which is in agreement with similar materials [58]. Moreover, a significant impact of Sn_0.8_Mn_0.2_S nanostructure loading on the activation energy of the synthesized nanocomposites is observed. E_ac_ decreased to 0.18 eV for the 1 wt.% of Sn_0.8_Mn_0.2_S incorporation, and then returned to rise again. Our findings reveal that the 1 wt.% of Sn_0.8_Mn_0.2_S/PVA nanocomposite film is optimal for electrical characteristics for such applications. The decrease in E_ac_ is caused by the minimization of the energy barriers relative to the segmental motion of the plain PVA polymer [54]. Similar findings are reported in Refs. [59,60,61]. For example, Ravi et al. [54] showed that 15 wt% of KIO_4_ loading in PVP produced a decrease in E_ac_ from 0.53 to 0.21 eV.

Taken together, the structural, optical, and electrical results point to a common origin. The XRD and SEM analyses show that Sn_0.8_Mn_0.2_S nanostructures progressively disrupt the crystalline order of the PVA host and, at high loadings, agglomerate into flake-like clusters. This same filler-induced disorder generates the localized sub-gap states responsible for the concentration-dependent narrowing of the optical bandgap and the enhancement in the whole optical constants described above. At low-to-moderate loadings (up to 1.0 wt.%), the nanostructures are well-dispersed within the PVA matrix and provide continuous pathways for charge-carrier hopping, explaining the parallel drop in electrical resistivity and activation energy up to these concentrations. For higher loadings, the agglomeration evidenced by SEM and XRD instead promotes carrier-trapping and vacancy-related centers, consistent with the partial recovery of both the resistivity and activation energy observed for the M5.0 and M10.0 samples. This consistent structure–property picture supports 1.0 wt.% Sn_0.8_Mn_0.2_S as the composition that best balances optical tunability and electrical performance for optoelectronic use.

## 4. Conclusions

Nanocomposite films, consisting of plain PVA films and Sn_0.8_Mn_0.2_S nanostructures, were equipped by a combination of a precipitation procedure for the filler and a solution casting method. FTIR spectroscopy confirmed the active integration between the Sn_0.8_Mn_0.2_S and PVA structure. The SEM investigations showed good dispersion for the low loadings of Sn_0.8_Mn_0.2_S nanostructures and an agglomerated flake-like morphology at high amounts. The XRD analysis reveals the semi-crystalline feature of the PVA film, with a characteristic reflection at 19.60°. The crystallinity characteristics increased with an increase in the Sn_0.8_Mn_0.2_S amount. The plain PVA film has two major absorption peaks at 209 nm and 276 nm, corresponding to the π→π* electronic transition. The transmittance of PVA decreased with cut-off edge redshifts to a longer wavelength due to Sn_0.8_Mn_0.2_S incorporation. The direct and indirect optical bandgap decreases from 5.36 eV and 4.95 eV for the plain PVA film to 1.94 eV and 1.32 eV for the 10.0 wt.% nanocomposite film, respectively. The refractive index increased from 1.69 to 2.33. Moreover, the dielectric constants and optical conductivity of the PVA film are significantly enhanced. The electrical measurements were performed in the temperature range of 300 to 400 K using a four-probe technique. The electrical resistivity of the PVA film decreased, and the activation energy decreased also from 0.39 eV to 0.18 eV up to a 1 wt.% loading of Sn_0.8_Mn_0.2_S nanostructures. Our findings reveal that the 1 wt.% of Sn_0.8_Mn_0.2_S/PVA nanocomposite film is optimal for electrical applications. These outcomes show that Sn_0.8_Mn_0.2_S nanostructures are a promising filler to control the structural, optical, and electrical performance of PVA film for optoelectronic and related applications.

## Figures and Tables

**Table 1:** Sample codes for the plain PVA and Sn_0.8_Mn_0.2_S/PVA nanocomposite films.

Sample	Sn_0.8_Mn_0.2_S Concentration (wt%)
Plain PVA	0
M0.1	0.1
M0.5	0.5
M1.0	1.0
M5.0	5.0
M10.0	10.0

**Table 2:** Activation energy (E_ac_) of Sn_0.8_Mn_0.2_S/PVA nanocomposite films (M0.1–M10.0).

Sample	E_ac_ (eV)
plain PVA	0.36
M0.1	0.35
M0.5	0.22
M1.0	0.18
M5.0	0.39
M10.0	0.31

## Data Availability

The original contributions presented in this study are included in the article. Further inquiries can be directed to the corresponding author.
